# The Use of Anabolic Agents in Catabolic States

**Published:** 2007-02-12

**Authors:** Robert Demling

**Affiliations:** Brigham and Women's Hospital, Harvard Medical School, Boston, MA

## Abstract

**Objective:** We plan to review the current problem of lean mass erosion in catabolic states, caused by injury and critical illness. This protein loss is driven by the hormonal imbalance and excess inflammation referred to as the “stress response to injury.” We then plan to provide the current concepts on the use of available anabolic agents to attenuate the excess catabolism. **Data Source:** The available published literature on the pathogenesis of acute catabolic states and the use of anabolic and anticatabolic agents, their indications, mechanism of action, and potential complications was reviewed. **Data Extraction:** The current understanding and experience of the available anabolic and anticatabolic agents as well as the rationale for the use of each anabolic agent are described. **Conclusion:** We conclude that the preservation of lean body mass (body protein) is extremely important in the management of critical care populations, as lean mass loss leads to severe morbidity and increased mortality. Essentially, all of the available anabolic agents stimulate protein synthesis and decrease protein breakdown, but all have different mechanisms of action. Adequate nutrition, especially protein intake, is essential for any anabolism to occur. Combined anabolic therapy also appears to be advantageous. Although controlling the inflammatory response would also be of major benefit in further controlling protein loss, effective and safe anti-inflammatory agents have not yet become clinically available for this purpose.

There is a very complex relationship between hormones, nutrition and protein synthesis, anabolism, or protein degradation. This is severely disrupted with bodily stress. The stress response to injury, including surgery or any significant illness, can be considered to be a maladaptive or autodestructive process.^1^–^3^ The body consumes itself, especially muscle, for energy instead of using body fat. This process is the result of an excessive catabolic activity due to both a hormonal imbalance and excess inflammation.^1^–^5^ A loss of lean body mass occurs, with the degree of loss corresponding with subsequent mortality and morbidity.

The initiating event, known as the “fright-flight” response, was added to the human genome thousands of years ago as a way of generating a surge of energy to deal with a short-term threat or injury.^1^ Since very little glucose, which is the primary source of immediate energy, is stored in the body, the most rapid source of energy is the components in body protein, namely, amino acids, which can be converted to glucose or used directly for energy.^1^–^4^

This response is activated immediately after a stress insult and peaks around 3 to 5 days postinsult. An inevitable loss of muscle and body protein occurs, which is deleterious to all bodily functions.^1^–^7^ Unfortunately, this catabolic process cannot be turned off until the injury or illness has resolved itself totally, even if adequate glucose is provided.^1^–^3^

The magnitude of this autodestructive response is in large part dependent upon the magnitude of the insult and the time course to complete recovery. The impact on the patient can be extremely harmful and can even prove fatal. So how can this process be controlled?

It is now well recognized that providing certain anabolic agents can help neutralize the net catabolism and restore hormonal balance in the stressed patient population, thereby significantly decreasing the catabolic response to “stress.”^8^,^9^ However, the degree of inflammation, which is also catabolic, has not yet been effectively altered with the possible exception of the anabolic hormone insulin-like growth factor-1 (IGF-1)/IGF-1-binding protein-3 (IGFBP-3).^5^–^7^

## THE STRESS RESPONSE TO INJURY OR ILLNESS

Essentially, any significant injury or illness will activate the catabolic “stress response,”^10^–^12^ and the outcome of any catabolic state is strongly influenced by the degree of net protein breakdown compared with the amount of net protein production or anabolism.^13^–^16^ This balance or imbalance will be determined by the magnitude and longevity of the catabolic state and the therapeutic modalities initiated to control this response. The metabolic abnormalities produced are fairly well described in Table 1.

The stress response is characterized by increased and protracted levels of the hormones epinephrine and cortisol, which increase energy demands beyond needs and cause increased protein breakdown, primarily for the production of excess glucose and for energy.^11^–^12^ In addition, levels of endogenous anabolic hormones, human growth hormone (HGH), and testosterone are decreased. The level of IGF also goes down, leading to a state of insulin resistance, insulin also being an anabolic hormone.^1^–^4^,^14^–^16^ This abnormal hormonal environment leads to a net increase in catabolism or protein degradation of muscle and visceral protein (Table 2).^14^–^20^

Inflammation, a component of any injury or infection, generates products, such as proinflammatory cytokines and oxidants, that will produce further protein degradation.^5^–^7^,^15^–^25^ Also, the production of important proteins, required for normal metabolism by the liver, is decreased in favor of what are known as “acute phase proteins,” which are primitive proteins with immune properties. The degree of acute-phase protein production typically corresponds with the magnitude of the inflammatory response.^26^–^28^

## CHANGES IN LEAN BODY MASS WITH A CATABOLIC STIMULUS

The lean body mass contains all the proteins present in the body as well as all the water. Two thirds of the protein is found in muscle and skin. The remainder is responsible for organ structure and function, immunity, enzyme activity, and any new tissue formation.^29^–^32^

As opposed to fat mass, which is basically a storage depot for available calories, there are no stored proteins; all have some significant physiological or metabolic function. Therefore, any net protein loss is harmful.^28^–^33^

Protein, instead of fat, becomes a major fuel source with “stress,” as seen in starvation, because of the abnormal hormonal and inflammatory environment. Breaking down protein over the long-term is maladaptive and autodestructive.^20^–^23^ Protein is also a very poor source of calories, generating only 4 calories per gram, yet breakdown of protein makes up more than 30% of the calories used after an injury or after any significant body insult. Loss of lean mass instead of fat will lead to significant complications (Table 3).

The complications of lean mass loss correspond to the amount and rate of lean body mass loss relative to total, assuming total to be not compromised.^30^,^33^

A lean body mass loss of 10%, which is quite common with critical illness, corresponds to increased complications.^30^–^33^ Protein loss will increase infection risk, which may prove fatal. With a large protein loss, 30% or greater, spontaneous wounds develop as loss of protein from the skin leads to skin breakdown with pressure. A loss of more than 40% of lean body mass is typically fatal as it leads to cell shutdown, which cannot be reversed (Table 4).^30^–^33^

It is also important to recognize that the restoration of body protein is at least 4-fold slower than the rate of loss although the use of anabolic agents can accelerate this restoration.^2^,^34^–^40^

## ANABOLIC AND ANTICATABOLIC AGENTS

It is now clear that controlling the catabolic state, by increasing anabolism and controlling inflammation, is essential to improving the outcome and decreasing complications in the severely injured and critical ill population. Therefore, the main frontier in critical care is to control both the excess protein loss from hormonal imbalance and the organ damage from inflammation.^19^–^25^ Controlling the injury state or illness is, of course, paramount. Providing optimum nutritional support is also essential to keep up with the increased energy (caloric) and protein demands as well as the increased intake of micronutrients. Typical required protein intake per day is 1.5 g/kg.^24^,^25^

The purpose of this review, however, is to focus on anabolic agents that can assist in controlling protein loss. Controlling the inflammatory response is something that is yet to be achieved.^21^–^26^

A number of anabolic and anticatabolic strategies are now available for clinical use. Several of these agents have been shown to be remarkably effective.^34^–^41^ Like any new treatment modality, the objective is to utilize the most effective agent or a combination of anabolic-anticatabolic agents with the fewest side effects. Either effect is recognized to be beneficial. Many of the anabolic agents also have anticatabolic properties, often due to down-regulation of cell cortisol receptors.^34^–^41^

In general, these agents are either amino acids or metabolites that stimulate protein synthesis or hormones with anabolic activity. All of the agents, currently available, have a specific mechanism of action either as a substrate or as an activator of specific cell functions, namely, protein synthesis (Table 5). Sufficient protein intake is essential to support any anabolic activity.

It is important to point out that none of the currently used anabolic agents have an effect on the important inflammatory catabolic component of the stress response with the possible exception of the compound IGF-1/IGF-BP3 (Table 6).

## AMINO ACID THERAPY

Specific amino acid therapy can lead to an increase in protein synthesis; however, there are no recognized effects on inflammation.^42^–^44^

### Glutamine

Glutamine is the main carrier of nitrogen between various tissues, including skeletal muscle, liver, intestines, and kidney. The liver uses glutamine as a preferred source of energy. Glutamine is also a precursor, along with cysteine, for the key intracellular antioxidant glutathione, which is produced in the liver and then exported to other organs, especially the lung. Enterocytes prefer to use glutamine instead of glucose as their primary energy source.

The availability of glutamine is now recognized as a rate-limiting step in muscle protein synthesis, and the rate of protein turnover in muscle depends in part on the availability of glutamine. In addition, there is a well-recognized glutamine deficiency state within 48 hours of a severe injury or illness and glutamine then becomes an essential amino acid. Increasing glutamine intake appears to have both anticatabolic and anabolic effects. Glutamine supplementation at the level of 0.5 mg/kg per day has been shown to significantly decrease mortality rates in major burn injury, significantly decrease infection rates, and increase protein synthesis in other high-risk critically ill populations^45^–^47^ (Table 7).

The major anabolic and anticatabolic property of glutamine is likely because of increased availability for protein synthesis in a postinjury deficiency state. Another potentially important anabolic action of glutamine is stimulating HGH release.^44^–^49^ The mechanism for this action remains unknown. There is not yet a unanimous opinion as to which critically ill patient populations benefit from glutamine supplementation. Certainly, it is effective in trauma patients but the effect is less clear in patients with sepsis.

Ornithine α-ketoglutatrate (OKG), a precursor of glutamine, is also reported to have anabolic activity. The mechanism of action of OKG is not clearly understood, but it appears to act by the enhanced secretion of anabolic hormones and the increased synthesis of metabolites, glutamine, polyamines, and arginine.

It is recommended that high-dose glutamine not be given in the presence of liver failure due to increased production of ammonia.

### Arginine

Arginine has been shown to have a wide variety of potentially beneficial metabolic effects in the injured or critically ill patient population. The most important pathway of arginine metabolism appears to be its conversion to orthinine in the liver, which is an obligatory precursor for protein synthesis (Table 8).^50^–^52^

Arginine supplementation, usually up to 20 g/d, has been reported to reduce weight loss and nitrogen loss and improve nitrogen retention and wound healing.^50^–^52^ The mechanism for this action is unclear. One mechanism may be stimulation of the release of HGH. In addition, there is clearly an increase in lymphocyte production and therefore an immune system stimulation effect. Improved wound healing, as evidenced by increased collagen deposition, has also been well described in experimental studies. Clinical data on healing or infection control is much less convincing.^50^–^52^ A possible complication of high-dose replacement may be increased production of nitric oxide, which has been reported to have both deleterious and beneficial effects. There are no studies on the advantages or disadvantages of arginine supplementation in critical illness at the present time, although there are a number of important products on the market with increased arginine content. More clinical studies, verifying the efficacy of arginine as an anabolic agent other than increased wound collagen deposition, still need to be performed.

### Hydroxy methyl butyrate

β-Hydroxy methyl butyrate (HMB) is a metabolite of the essential amino acid leucine. It has been shown, in a number of clinical trials, to decrease catabolism in normal man and in the elderly after exercise. The mechanism of action appears to be related to the fact that leucine depletion, during stress, increases catabolism and providing the HMB metabolism, blocks this response. In addition, HMB has been shown, in several clinical trials, to increase the restoration of lean mass in conjunction with exercise, felt to be the result of its anticatabolic effect.^53^–^55^ HMB is available for clinical use as a powder, with the recommended dose being 1.5 g every 12 hours.

### Combined amino acids

Glutamine, arginine, and HMB were combined in a nutritional supplement (JUVEN) that showed a decrease in catabolism and an increase in lean mass in catabolic states (HIV, cancer, and elderly weight loss) in 3 randomized controlled studies.^56^,^57^ It is important to note that all 3 components work through different metabolic pathways. However, the role of each amino acid in the anabolic actions of the combined product is not known.

## ANABOLIC HORMONE THERAPY

### Insulin

Insulin is a naturally occurring endogenous polypeptide hormone best known for controlling blood glucose levels by increasing glucose uptake at the cell level. Insulin also has potent anabolic and anticatabolic properties and has been shown in a number of trials to increase protein synthesis, especially when given in increased concentration to burn and trauma patients (Table 9).^58^–^66^

Its mechanism of action is complex but mainly involves transport of amino acids, glucose, and fat into the cell while decreasing the efflux of amino acids from the cell.^61^

Its anticatabolic effect relates to a decrease in proteolyses. The anabolic activity is mainly seen in the protein content of muscle and skin in the lean mass compartment. The anabolic response to insulin decreases with aging while most other anabolic agent activity is not age related. Increased re-epithelialization of skin graft donor sites was reported in one clinical trial in burn patients. Several animal studies have demonstrated increased collagen production with insulin and increasing the level of insulin administered to mice with diabetes improved all phases of healing. However, the effects of insulin on wound healing have not been well studied in man.

The major complication with its use as an anabolic agent is hypoglycemia, requiring rigorous monitoring of glucose levels. Also, because of its short half-life, a continuous parenteral insulin infusion is especially utilized. There are no recognized effects of insulin on the inflammatory phase of the stress response. Insulin will also cause fat production in liver if excess glucose is also present.

### Insulin-like growth factor-1

IGF-1 is a naturally occurring large polypeptide that has hormone-like properties. IGF-1, also known as somatomedin-C, has metabolic and anabolic properties very similar to those of insulin (Table 10).^67^–^72^

IGF-1 is produced by a variety of wound cells, such as fibroblasts and platelets. The main source of production is the liver where IGF synthesis is initiated by HGH. The IGF receptor on the cells is expressed in many different tissues and active peptide is bound, in plasma, by IGF-binding proteins. Its production is decreased in “stress,” especially sepsis.^67^ Anabolic activity is noted when IGF-1 is provided by continuous infusion.^68^–^72^ However, the response is difficult to separate from that of increases in endogenous insulin or HGH. There are no clinical studies showing anti-inflammatory activity with IGF-1. Also, an IGF-1 infusion loses its anabolic activity with long-term use.

The attenuation of stress-induced hypermetabolism is a favorable property of IGF-1. The clinical trials using an IGF-1 infusion have focused on demonstrating increased anabolic activity. Increased protein synthesis and nitrogen retention has been reported in burns, head injury, and HIV-induced catabolic states.

The major problem with its use is the risk of hypoglycemia (low glucose). Also problematic is the need for a continuous intravenous infusion, requiring that glucose levels be monitored. Low-dose infusions are not effective. The ideal dose has not yet been determined.

### Insulin-like growth factor-1 bound to IGF-1-binding protein-3

Binding of IGF-1 to its major binding protein IGFBP-3 results in new and very advantageous properties compared to those of IGF-1 alone (Table 11).

As expected, many properties remain similar to those of IGF-1 and insulin. However, the half-life is increased from minutes to more than 12 hours. Exogenous IGF infusion, over time, appears to lead to an attenuation of its anabolic effects. This attenuation is not seen with the exogenous administration of the IGF-1/IGFBP-3 complex. There is a significant increase in protein synthesis (anabolism) and anticatabolic properties persist and remain constant with long-term administration. Interestingly, the anabolic effects of IGF-1/IGFBP-3 increase as the catabolic stimulus increases. Increased wound healing has also been demonstrated, much like that for IGF-1.^73^–^80^

Of major importance is the effect of this hormone and protein complex on excess inflammation, an important component of the “stress” catabolic response. IGF-1/IGFBP-3 has been shown in burn patients, who have a profound catabolic and the systemic inflammatory response, to decrease the magnitude of both catabolism and inflammation.^77^–^80^ This effect was identified by a decrease in the levels of protein products in the acute phase.^77^–^80^ Levels of important proteins, normally suppressed, such as prealbumin and albumin, were increased.

In addition, there was a decrease in proinflammatory cytokines, which are activators of inflammation, resulting in a better balance between proinflammatory and anti-inflammatory cytokines. Remarkably, but not unexpectedly, this attenuation of the inflammatory response corresponds with improved cardiac, liver, and renal function.^80^ It would be anticipated that attenuation of inflammation would improve organ function as organ failure is the typical response to autodestructive inflammation.^23^–^29^ Organ failure is a major cause of mortality in severe catabolic states.^23^–^29^,^79^

The other beneficial effect of this complex as opposed to other anabolic agents is its effect on normalizing blood glucose levels. Both low and high blood glucose levels, seen with the use of some other anabolic agents, have been well documented to be very deleterious.^73^–^80^

### Testosterone

Testosterone, whose basic structure is a steroid ring, is the natural endogenous androgen. Testosterone is synthesized primarily in the testicles in males and by the ovaries and adrenal gland in females. Testosterone acts on the cells' androgenic receptors found mainly in skin, muscle, and male sex glands.

It has both androgenic and masculinizing properties and anabolic or protein synthesis properties (Table 12). The importance of testosterone is evidenced by the complications seen with low testosterone level, which include sarcopenia or lost lean mass, increased rate of development of osteoporosis, anemia, thinning of skin, impaired wound healing, and weakness (Table 13).^81^–^86^

Testosterone levels decrease with any severe stress. Testosterone replacement is essential in hypogonadal states to avoid further lean mass loss and the other complications of low testosterone levels. Replacement is typically done by depot injection.

However, beyond replacement therapy, testosterone is not used as an anabolic agent as it has relatively weak anabolic activity compared to its analogs and its androgenic side effects can become problematic.^82^,^83^ Testosterone has no effect on the inflammatory process or on glucose metabolism. The major complications with its use are a decrease in high-density lipoproteins, some fluid retention, and endrogenic effects.

### Oxandrolone (testosterone analog)

Anabolic steroids refer to the class of drugs produced by modification of testosterone.^85^–^93^ These drugs were developed in order to take clinical advantage of the anabolic effects of testosterone while decreasing the androgenic side effect of the naturally occurring molecule. Modifications were made in the steroid ring because of the short half-life of testosterone and its masculinizing properties. Modifications included a 17-α-methyl derivative for oral use and a 17-ß-ester configuration for parenteral use. These changes markedly increased its half-life and decreased its androgenic properties.

The mechanisms of action of testosterone analogs are also through activation of the androgenic receptors found in highest concentration in myocytes and skin fibroblasts. Some populations of epithelial cells also contain these receptors. Androgenic receptors were first isolated in the 1960s.

Stimulation of these receptors leads to a decrease in the efflux of amino acids and an increase in the influx into the cell. Activation of intracellular DNA and DNA polymerase also occurs with androgenic receptor stimulation. A decrease in fat mass is also seen because of the preferential use of fat for fuel. There are no metabolic effects on glucose production.

All anabolic steroids increase overall protein synthesis and new tissue formation, as evidenced by an increase in skin thickness and muscle formation. All these agents also have anticatabolic activity, decreasing the protein degradation caused by cortisol and other catabolic stimuli.^1^ In addition, all anabolic steroids have androgenic or masculinizing effects (Table 14).

Oxandrolone is a synthetic anabolic steroid with potent anabolic and anticatabolic activity with minimal androgenic (masculinizing) properties.^85^–^93^ It acts on cells' androgenic receptors, found mainly in the lean mass compartment, to increase protein synthesis.

Oxandrolone, a modified form of testosterone, is the only FDA-approved anabolic steroid used for restoring lost body weight. The safety advantage of oxandrolone is that it is cleared by the kidney rather than the liver so hepatoxicity, which is a major problem with other anabolic steroids, is less of an issue. Its anabolic activity is approximately 10 times that of testosterone and its androgenic activity is one tenth that of testosterone. Currently, this agent is being used clinically not only to restore lost lean mass but also to preserve lean mass loss in catabolic states. Its half-life is 9 to 12 hours. Oxandrolone is given orally twice a day, usually 10 mg per dose. It has been shown to decrease net catabolism in a number of catabolic states, burns being the most significant. Anabolism is increased in both the acute state and during the recovery period (Table 15).

Testosterone analogs act only on androgenic receptors found only in the lean body mass compartment.^85^ There are no effects on metabolism other than protein synthesis. Testosterone analogs cannot be used in the presence of androgenically sensitive^85^ tumors, which include prostate cancer and male breast cancer. Oxandrolone also increases sensitivity to Coumandin, necessitating adjustment in its dosage.

Studies indicate that anabolic activity is markedly increased in injured men, elderly with and without wounds, steroid-dependent patients, cachexia, HIV, chronic obstructive pulmonary disorder, and cancer chemotherapy with weight loss.^87^–^93^ Adequate protein intake is required.

### Human growth hormone

HGH is a potent endogenous anabolic hormone that is also anticatabolic, acting on a specific HGH cell receptor. HGH is a large polypeptide with a number of binding proteins and cell-binding sites. Starvation and intensive exercise increase HGH production. Severe, acute, or chronic illness decreases HGH levels.^94^,^95^

Clinical studies have in large part focused on the systemic anabolic and anticatabolic actions of HGH. Populations in which HGH has been shown to have beneficial effects include those with severe burn and trauma, those with HIV infection with wasting, and frail elderly adults. In addition, HGH is being used to slow down the aging process. Increase in lean mass, muscle strength, and immune function has been documented in clinical use. HGH is approved only for use in children of short stature and is an orphan drug when used for improving protein synthesis. Increased anabolic activity requires ingestion of a high-protein, high-energy diet.^96^–^100^

As to any direct wound healing effects, skin is a target tissue for HGH, both directly through HGH receptors on the surface of the epidermal cells and indirectly through the action of IGF-1. Exogenously administered HGH has been shown to increase skin thickness in normal humans.^48^ Other effects on the wound include increased rate of re-epithelialization of skin graft donor sites in adults and children with severe burns or trauma (Table 16). In addition, HGH has been shown to increase wound collagen content, granulation tissue and wound tensile strength, and the local production of IGF-1 by fibroblasts. These data are derived mainly from animal studies.

HGH when provided typically binds with one of its binding proteins. HGH has a number of metabolic effects, the most prominent being its anabolic activity but insulin resistance also occurs, which often leads to hyperglycemia, which will increase morbidity. A positive metabolic response is the increased use of fat for fuel. Another negative effect is the increase in metabolic rate (10%–15%), which occurs with the typical dose of HGH (10 mg twice a day in an adult) or 0.2 to 0.3 mg/kg per day. HGH must be given parenterally.

HGH has been used in the clinical setting for at least 25 years for its anabolic and anticatabolic activity.^96^–^100^ Recently, evidence has come up that HGH can be harmful to certain critical care populations.^101^,^102^ This is likely the result of the inevitable hyperglycemia, which has now been shown to markedly increase morbidity and mortality in the critically ill. In addition, the increase in metabolic rate can be deleterious in an already hypermetabolic patient.^101^,^102^

## CONCLUSION

The “stress” or “fright-flight” response occurs with any significant injury, including surgery, infections, or critical illness. This genetically programmed response, which may have been beneficial for short-term insults, has become maladaptive and autodestructive in modern man because of the longer time course and more severe insults, which are managed today with modern medicine.

This response is a combination of an abnormal hormonal imbalance and excessive injury/insult-induced inflammation. Increase in the levels of catabolic hormones (cortisol and epinephrine) and decrease in the levels of anabolic hormones (HGH and testosterone) leads to increased protein breakdown and decreased protein synthesis.

In addition, inflammation, driven by excessive proinflammatory cytokines, also causes catabolism (protein breakdown), resulting in infections, organ damage, and death.

Managing the catabolic, hormonal, and excessive inflammatory state is becoming a primary focus of new advances in critical care. There are a number of anabolic and anticatabolic agents currently being used, in critical care, that have beneficial properties, by improving overall net anabolism. It is clear that all these agents are effective in the catabolic states of injury and illness.

The anabolic amino acids glutamine, arginine, and HMB all have significant anabolic and anticatabolic activity. Glutamine clearly has the most potent properties.

The anabolic hormones currently available include insulin, IGF-1, and IGF-1/IGFBP-3, which is IGF-1 bound to the IGF-1-binding protein 3, testosterone and its analogs, and HGH.

The anabolic amino acids are quite safe and do increase anabolism in stress states. There are also a number of anabolic and anticatabolic hormones being used. All of them decrease but do not eliminate catabolism. The reason is that inflammation is not controlled. Of interest is the finding that the anabolic agent IGF-1/IGFBP-3 appears to not only increase anabolism but also decrease the inflammatory response. In addition, glucose dysregulation is not seen. Hypoglycemia and hyperglycemia are also seen with insulin, IFG-1, and HGH, respectively. This problem can be controlled with adequate monitoring of glucose levels.

Overall, current clinical data would indicate that anabolic therapy can be safely integrated into the management of severe injury and critical illness and should result in improvement in outcome.

## Figures and Tables

**Table 1:** Metabolic abnormalities due to the “stress response” of injury

Increase in levels of catabolic hormones (cortisol and catechols) Decrease in levels of anabolic hormones (human growth hormone and testosterone) Increase in levels of proinflammatory cytokines Decrease in levels of anti-inflammatory cytokines Increase in acute-phase protein production Marked increase in metabolic rate (with increased energy initialization) Sustained increase in body temperature Rapid skeletal muscle breakdown with amino acid use for both energy source and gluconeogenesis Insulin resistance, resulting in an increase in blood glucose levels Unresponsiveness of catabolism to nutrient intake (feeding)

**Table 2:** Actions of key hormones in metabolism

	**Catechols**	**Cortisol**	**Insulin**	**Human growth hormone**	**Testosterone**
Gluconeogenesis	↑	↑	↑	↑	…
Glycogen formation	↓	↓	↓	↓	…
Glycogenolysis	↑	↑	↓	↑	…
Lipogenesis	↓	↑	↑	↓	↓
Lipolysis	↑	↓	↓	↑	↑
Ketone production	↓	↓	↓	↑	…
Protein synthesis	↑	↓	↑	↑↑	↑
Proteolysis	↑	↑	↓	↓	↓

**Table 3:** Hormonal response to stress and starvation*

	**Starvation**	**Stress**
Catechols	↓	↑ ↑
Cortisol	↓	↑ ↑
Insulin	↓	↑
Glucagon	↓	↑
Human growth hormone	↑ ↑	↓
Testosterone	↓	↓

*With starvation, an adaptive hormonal response is present, preserving lean mass and energy, while a maladaptive catabolic state occurs with the “stress response” activated by any wound.^12^,^31^

**Table 4:** Complications relative to loss of lean body mass

Lean body mass,* % loss of total	**Complications (related to lost lean mass)**	**Associated**
10	Impaired immunity, increase in infection	10
20	Decrease in healing, weakness, infection	30
30	Too weak to sit, pressure sores, pneumonia, no healing	50
40	Death, usually from pneumonia	100

*The complications correspond with the degree of loss relative to total lean body mass, assume no preexisting loss as is often present especially in the elderly.

**Table 5:** Available anabolic agents

**Amino acids or metabolites**
Glutamine
Arginine
Hydroxy methyl butyrate
**Anabolic hormones**
Insulin
Insulin-like growth factor-1 (IGF-1)
IGF-1 with IGFBP-3*
Testosterone
Anabolic steroids
Human growth hormone

*IGF-1 bound to IGF-1-binding protein 3.

**Table 6:** Effects of available anabolic agents

**Agent**	**Anabolic**	**Anticatabolic**	**Inflammation**	**Blood glucose**
Glutamine	↑	↑	…	…
Arginine	↑	…	…	…
Hydroxy methyl butyrate	…	↑	…	…
Insulin	↑	↑	…	↓
Insulin-like growth factor-1 (IGF-1)	↑	↑	…	↓
IGF-1 plus IGFBP-3*	↑	↑	↓	Maintains
Testosterone	↑	↑	…	…
Oxandrolone	↑	↑	…	…
Human growth hormone†	↑	↑	…	↑

*IGF-1 bound to IGF-1-binding protein 3.

^†^Human growth hormone + β-hydroxy methyl butyrate.

**Table 7:** Metabolic effects of glutamine

A deficiency state occurs with “stress” Primary nutrient source for gut mucosa Precursor of glutathione (antioxidant) Anticatabolic activity at the muscle cell level Anabolic activity with supplementation at 0.5 mg/kg per day Improves nitrogen retention Stimulates human growth hormone release (anabolic activity) Decreases infection rates Improves survival after severe injury Increases ammonia in liver failure

**Table 8:** Metabolic effects of arginine

Levels decrease with “stress” Increases nitric oxide production Reduces nitrogen loss Reduces weight loss Stimulates lymphocyte activity May stimulate release of human growth hormone

**Table 9:** Metabolic effects of insulin

Endogenous anabolic hormone Activity decreased with “stress” Increases transport of amino acid into cells Decreases loss of amino acid from cells Stimulates protein synthesis in catabolic states Decreases protein breakdown in catabolic states Excess amounts can lead to hypoglycemia

**Table 10:** Metabolic effects of insulin-like growth factor-1

Endogenous anabolic hormone Levels decrease with “stress response” Production by many cells, especially in the liver Production dependent on adequate levels of human growth hormone Actions much like those of insulin Increases protein synthesis Known to directly stimulate wound healing Extremely short half-life when binding protein is not attached Can produce problematic hypoglycemia Requires continuous infusion with monitoring of glucose levels Decreased efficacy with long-term infusion

**Table 11:** Metabolic effects of IGF-1/IGFBP-3

Endogenous anabolic hormone Levels decrease with injury/infection/stress Production directly correlated with human growth hormone levels Increases protein synthesis (anabolism) Decreases protein breakdown (catabolism) Attenuates proinflammatory response Attenuates the acute phase response to “stress” Improves production of essential proteins by the liver Improves organ function in a catabolic state Maintains normal glucose

**Table 12:** Metabolic effects of testosterone

Endogenous anabolic hormone Levels decrease with “stress” response Modest anabolic activity compared to that of its analogs Rapidly metabolized by the liver Levels decrease with increasing age Decreased levels of testosterone cause lean mass loss No effects on glucose metabolism Androgenic (masculinizing) side effects include male sex gland development, male pattern of hair, and mood

**Table 13:** Effect of decreased levels of testosterone (the hypogonadal state)

Lean mass loss Thinning of skin Increase in fat mass Impaired wound healing Decrease in physical and psychological masculinizing properties Osteoporosis

**Table 14:** Anabolic activity of 17 methyl derivatives

**Agent**	**Androgenic: Anabolic activity**	**Indication**	**Hepatoxicity**
Testosterone	1:1	Hypogonadism	
Nandrolone	1:4	Anemia	Moderate to severe
Oxymetholone	1:3	Anemia	Severe
Oxandrolone*	1:16	Loss of body weight from injury or infection	Mild, rare

*The anabolic steroid oxandrolone is the only approved drug for restoration of lost body weight and lost lean mass.

**Table 15:** Metabolic effects of oxandrolone

Testosterone analog Stimulates protein synthesis only in the lean mass compartment No effect on glucose metabolism Increases use of fat for fuel Marked attenuation of catabolism Increases anabolic activity Rapid restoration of lean body mass Cannot be used in the presence of an androgenic tumor Increases coumadin sensitivity

**Table 16:** Metabolic effects of human growth hormone

Endogenous anabolic hormone Decreased production with “stress” Increases cell uptake of amino acids Increases protein synthesis (anabolism) Decreases catabolism Increases release of insulin-like growth factor-1 Increases insulin requirements Increases use of fat for fuel Increases metabolic rate (10%–15%) Produces insulin resistance, often leading to hyperglycemia May increase mortality in subpopulations of critically ill patients No anti-inflammatory effects
